# Optimizing Sleep Disorder Management in Hospitalized Patients: Practical Approach for Healthcare Providers

**DOI:** 10.1002/brb3.70282

**Published:** 2025-02-09

**Authors:** Ghazal Roostaei, Niloofar Khoshnam Rad, Besharat Rahimi, Alireza Asgari, Shima Mosalanejad, Hossein Kazemizadeh, Maryam Edalatifard, Hamidreza Abtahi

**Affiliations:** ^1^ Thoracic Research Center, Imam Khomeini Hospital Complex Tehran University of Medical Sciences Tehran Iran; ^2^ Departrment of Internal Medicine, Faculty of Medicine Tehran Medical Sciences, Islamic Azad University Tehran Iran

**Keywords:** hospitalized patients, management, optimization, review, sleep

## Abstract

**Purpose:**

To provide a comprehensive review of sleep disturbances in hospitalized patients, focusing on a case‐based approach to illustrate the multifaceted nature of this clinical challenge.

**Method:**

An extensive review of related literature was conducted to determine the common causes of sleep disturbances in hospitalized patients, such as environmental, medical, psychological, and physiological factors. The case of Mrs. Z was used to illustrate how these factors interact in a clinical setting.

**Findings:**

The study revealed a high prevalence of sleep disturbances in hospitalized patients, which can lead to significant adverse outcomes. A multidisciplinary approach involving physicians, nurses, pharmacists, and other healthcare professionals is essential to effectively manage sleep disorders due to the interplay of various factors. Nonpharmacological interventions are fundamental to a comprehensive sleep management plan. Pharmacotherapy may sometimes be necessary to improve sleep quality and duration.

**Conclusion:**

Health professionals can significantly enhance the sleep quality of hospitalized piatients by understanding the value of sleep and providing evidence‐based strategies for improvement. In return, this improves patient outcomes, reduces healthcare costs, and advances general patient satisfaction.

## Graphical abstract

Hospitalized patients often experience sleep disturbances due to various factors, leading to negative health outcomes. A multi‐faceted approach, including environmental modifications, pharmacological interventions, and non‐pharmacological strategies, can significantly improve sleep quality and patient outcomes.

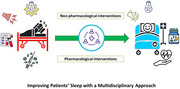

1

**Case presentation**

*Mrs. Z, a 71‐year‐old retired teacher with a history of heart failure and atrial fibrillation, was hospitalized for acute decompensation. During her stay, she experienced significant sleep disturbances. The noisy hospital environment, frequent interruptions, and lower back pain when lying flat disrupted her sleep. Additionally, anxiety, a newly prescribed beta‐blocker (metoprolol tartrate 25 mg twice daily), chronic insomnia, and untreated restless legs syndrome further exacerbated her sleep difficulties*.

**Table:** 

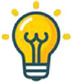	**How can healthcare providers effectively address Mrs. Z's multifaceted sleep disturbances?**

## 2 Introduction

Sleep is a fundamental biological process essential for maintaining both physical and mental health. It plays a vital role in regulating brain function, immune responses, metabolic stability, hormonal balance, and cardiovascular health (Mansour and Knauert 2022). Disruption to sleep affects multiple organ systems, harms overall health, slows recovery, and prolongs hospital stays (Vaishnav et al. 2023). Despite its importance, sleep disturbances are widespread in hospitalized patients due to environmental factors, frequent care‐related interruptions, and the physiological effects of acute illness (Burger et al. 2022).

The matter of poor sleep during or after hospitalization is not limited to critical care patients (Hillman et al. 2023). A large‐scale study of general ward patients found that hospitalized patients experienced significantly reduced total sleep duration, more frequent nocturnal awakenings, and earlier wake times (Wesselius et al. 2018). Common sleep disorders in hospitalized patients include insomnia and circadian rhythm disturbances. Less frequently, conditions such as sleep‐disordered breathing, hypersomnia, and restless leg syndrome are also reported (Stewart and Arora 2022).

**FIGURE 1:**
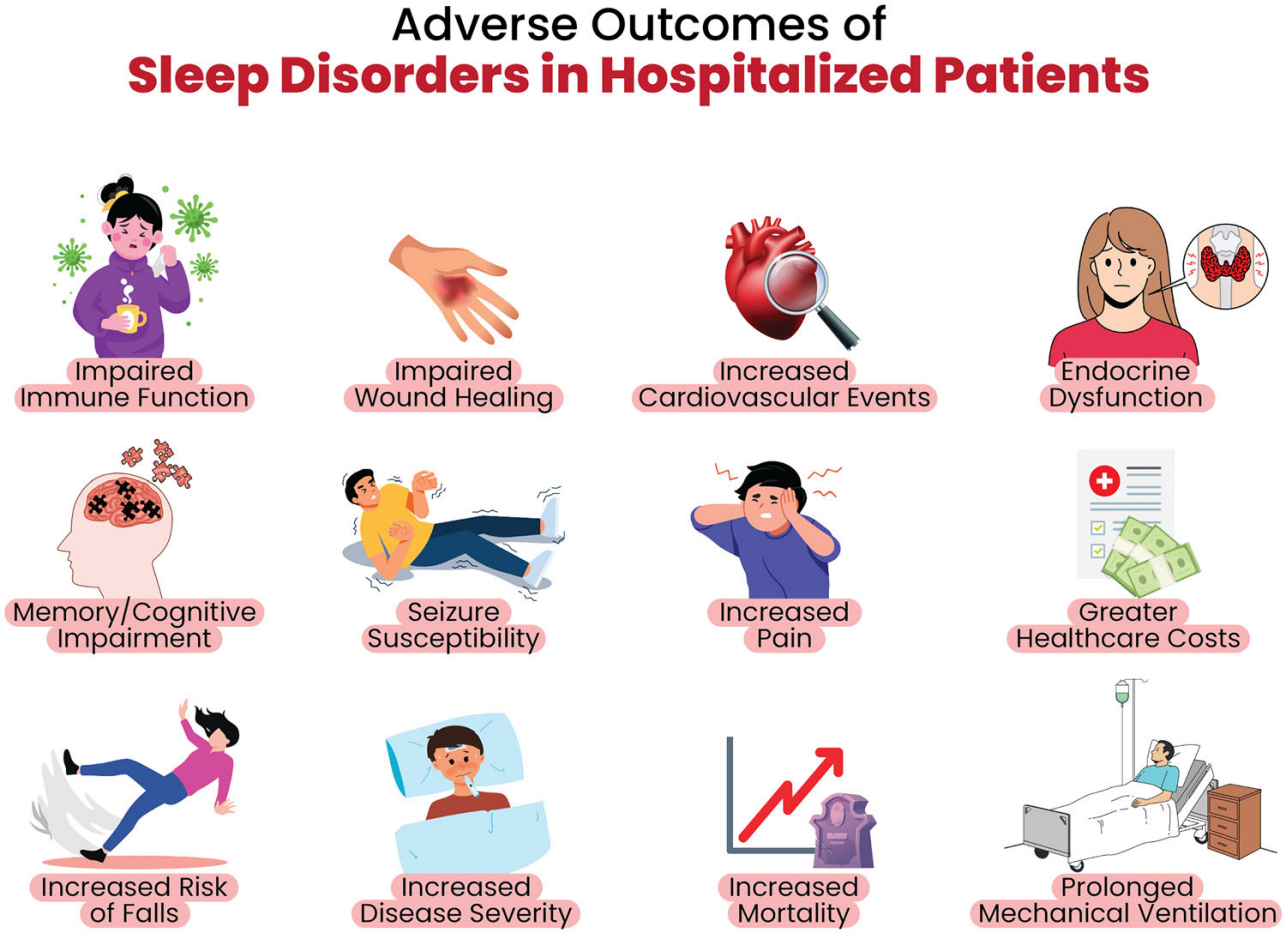
Adverse outcomes of sleep disorders in hospitalized patients.

Poor sleep quality may have significant short‐term effects. It can increase pain sensitivity, cause anxiety, and contribute to delirium. In addition, it impairs immune function and delays wound healing (Medic, Wille, and Hemels 2017; Locihová et al. 2023; Besedovsky et al. 2019; Marconi et al. 2024). Long‐term consequences of poor sleep quality include higher rates of hospital readmissions, reduced quality of life, and increased healthcare costs (Medic, Wille, and Hemels 2017; Isaia et al. 2011). These adverse outcomes are summarized in Figure 1. Despite these significant impacts, sleep disorders in hospitalized patients are often underdiagnosed and inadequately treated due to limited awareness among healthcare providers (Almeneessier et al. 2018).

This review aims to provide a comprehensive exploration of sleep disorders in hospitalized patients. It examines the physiological and environmental challenges to sleep in these settings and outlines evidence‐based approaches for assessment, diagnosis, and management. Emphasizing a multidisciplinary approach involving collaboration between physicians, nurses, pharmacists, and other healthcare professionals (Cicolin and Ferini‐Strambi 2023), the review seeks to bridge the gap in care and improve patient outcomes.

## 3 Sleep Disorders and Consequences in Hospitalized Patients

Normal sleep consists of cycles of non‐rapid eye movement (NREM) and rapid eye movement (REM) sleep, which follow a predictable pattern throughout the night. Each cycle typically lasts 90 to 120 min and repeats four to five times per night (Yetton et al. 2018). NREM sleep includes three stages (N1, N2, and N3), which are essential for restorative processes such as tissue repair and immune function. REM sleep is associated with vivid dreaming, muscle paralysis, and brain activity similar to wakefulness, playing a critical role in memory consolidation and emotional regulation. The distribution of sleep stages changes throughout the night, with a higher proportion of NREM sleep, particularly stage N3 (“deep sleep” or “slow‐wave sleep”), occurring in the first half of the night, and more REM sleep in the second half (Berry et al. 2013).

Hospitalization disrupts normal sleep patterns, leading to fragmented sleep. This often results in reduced slow‐wave and REM sleep, increased daytime sleepiness, and lower sleep efficiency (Hillman 2021). Patients may experience prolonged sleep latency, frequent awakenings, and early morning arousals, all contributing to poorer sleep quality (Burger et al. 2022). These disruptions can signal underlying sleep disorders related to the sleep‐wake cycle, circadian rhythms, or other physiological processes (Patel et al. 2020).

Poor sleep quality in hospitalized patients is associated with numerous adverse consequences (Figure 1) (Medic, Wille, and Hemels 2017). Circadian disruptions can exacerbate disease severity, stimulate inflammatory processes, impair treatment response, and decrease survival (Fishbein et al. 2021). Sleep deprivation is associated with cognitive, metabolic, cardiovascular, pulmonary, autonomic, and endocrine dysfunction. Its effects include fatigue, reduced secretion of growth hormone, disorientation, and impaired glucose metabolism (DePietro et al. 2017). Insomnia has been linked to an increased risk of high blood pressure and tachycardia, even following just one night of poor sleep (Miller et al. 2019; Oseni et al. 2024). Sleep fragmentation further contributes to cardiovascular instability, including increased blood pressure variability (Chang et al. 2020).

Emotional and cognitive impacts of disrupted sleep include depression, memory impairment, and diminished attention and learning capacity (Chang et al. 2020; Blackwell et al. 2024). Immune function is particularly affected, with reduced activity of natural killer cells and disrupted cytokine regulation (Chang et al. 2020). Critically ill patients are particularly at heightened risk, as sleep disturbances can lead to prolonged mechanical ventilation and increased mortality (Showler et al. 2023).

Pre‐existing sleep disorders, such as obstructive sleep apnea (OSA), often deteriorate during hospitalization. Patients with OSA face higher risks of postoperative complications, including oxygen desaturation, prolonged intensive care unit (ICU) stays, and greater mortality. Pregnant women with OSA also have an increased risk of postpartum maternal morbidity. OSA is associated with chronic conditions such as heart failure, coronary artery disease, and stroke, highlighting the need for tailored management during hospitalization (Stewart and Arora 2022).

### 3.1 Factors Contributing to Sleep Disturbances in Hospitalized Patients

**FIGURE 2:**
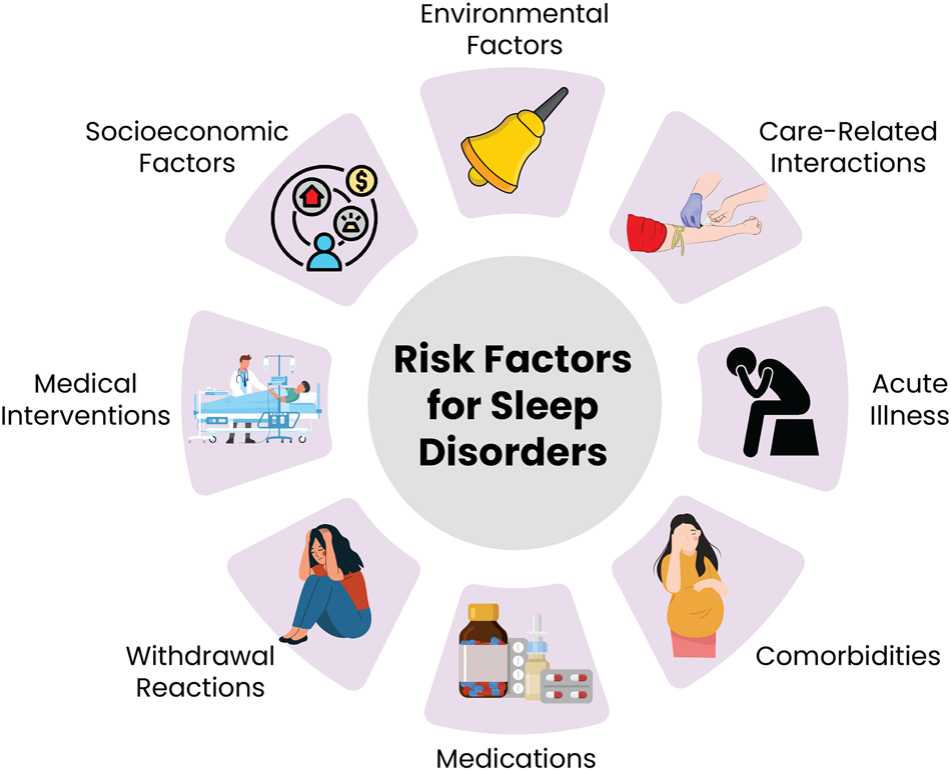
Risk factors for sleep disorders in hospitalized patients.

Several factors contribute to sleep disturbances in hospitalized patients, including excessive lighting at night, inadequate lighting during the day, noise, frequent interruptions for vital sign monitoring and medication administration, and the stress of an unfamiliar environment (Figure 2) (Koshy et al. 2024).

### 3.2 Environmental Factors

Hospital environments are often noisy, brightly lit, and subject to frequent interruptions, all of which can disrupt sleep patterns (Kulpatcharapong et al. 2020). Noise from medical equipment, alarms, staff conversations, and other sources, such as phones, televisions, and pagers, is commonly cited as a primary cause of sleep disturbance. In fact, hospital noise levels frequently exceed those recommended by the World Health Organization for optimal sleep quality (Smith et al. 2022). Polysomnographic studies in ICU have shown that noise contributes to 10–20% of arousals (Smith et al. 2022). Likewise, lighting, both excessive at night and inadequate during the day, further affects sleep. Nighttime light exposure in the ICU is often low (less than 2 to 4 lux), which typically supports sleep, but daytime light levels are often suboptimal (70 to 80 lux), resembling a dimly lit environment that can disrupt circadian rhythm synchronization. This issue is not limited to the ICU; low daytime light levels have also been observed in general medical wards (Pamuk and Turan 2022; Kamdar et al. 2012).

*For Mrs. Z, environmental factors such as persistent noise from alarms and staff conversations, as well as partial nighttime lighting, likely contributed to her difficulty falling asleep and staying asleep*.

**To improve Mrs. Z's sleep environment, we can**:

**Table:** 

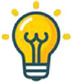	• **Reduce noise**: Use earplugs, minimize staff noise, and adjust equipment volume. • **Optimize lighting**: Provide low‐level, warm‐toned light at night and increase natural light exposure during the day.

## 4 Care Interactions

Acute illness care, particularly in ICUs, involves frequent patient interactions such as vital sign checks, blood draws, diagnostic tests, medication administration, phlebotomy, wound care, and hygiene tasks. These interactions, which increase in frequency with illness severity, can significantly disrupt sleep and circadian rhythms (Wesselius et al. 2018; Daniel et al. 2024). Studies report up to 40–50 interactions per patient per night, with hourly interruptions in some cases (Meyer et al. 1994). Between 5% and 15% of nocturnal interventions have been identified as potential modifiable causes of arousal, with up to 7% of sleep disruptions linked to patient care interruptions (Gabor et al. 2003).

*Mrs. Z's sleep was likely fragmented by frequent nighttime interruptions from routine checks, medication rounds, and early morning blood draws, disrupting her natural sleep cycle*.

**To minimize interruptions, we can**:

**Table:** 

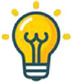	• **Cluster care**: Group routine checks and medication administration into specific time windows. • **Optimize scheduling**: Schedule procedures like phlebotomy during less disruptive times. • **Prioritize urgent needs**: Focus on essential interventions and avoid unnecessary disturbances.

### 4.1 Acute Illness

Hospitalized patients, particularly those with chronic illnesses or undergoing surgery, often experience pain, anxiety, or delirium. These factors can negatively impact sleep quality. Untreated or poorly managed pain can make it difficult to fall or stay asleep. It also reduces slow‐wave and REM sleep, leading to increased daytime sleepiness (Catalá et al. 2023; Miettinen et al. 2022). Anxiety and stress can cause hyperarousal, which disrupts sleep patterns and contributes to insomnia (Tan et al. 2019).

*Physical discomfort from lower back pain, exacerbated by heart failure symptoms, likely contributed significantly to Mrs. Z's sleep disturbances*.

**To improve her comfort**:

**Table:** 

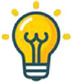	• **Pain management**: Collaborate with pain management specialists to address her back pain effectively. • **Positioning**: Use pillows to support her back and alleviate pressure points. • **Frequent repositioning**: Encourage her to change positions regularly to prevent discomfort. • **Physical therapy**: Consult with a physical therapist to develop a personalized exercise plan to strengthen back muscles and improve posture.

### 4.2 Surgical Patients

Surgical patients are particularly vulnerable to sleep disturbances due to the frequent need for monitoring, phlebotomy, and dressing changes overnight. In addition, concerns about moving monitors and tubes or maintaining specific positions due to postoperative pain can further disrupt their sleep (Hou et al. 2022). While anesthesia contributes to postoperative sleep disruption, it is not the primary cause. Anesthesia, whether regional or general, can reduce REM sleep, shorten total sleep time, and increase sleep fragmentation (Rampes et al. 2020). However, postoperative pain is a more significant factor. As pain intensity and frequency are heightened after surgery, it can significantly disrupt sleep, exacerbating disturbances (Rampes et al. 2020). Therefore, effective management of postoperative pain is crucial for improving sleep quality in surgical patients.

### 4.3 Critically Ill Patients

Circadian dysrhythmias are common in ICU patients and can negatively affect their prognosis. However, the complex interactions of pathophysiological, pathopsychological, pharmacological, and environmental factors contributing to sleep and circadian rhythm disturbances in this population are not fully understood. Despite growing awareness, optimizing sleep remains a low priority in the ICU due to the critical nature of patient conditions (Nilius et al. 2021).

Ventilation, both invasive and noninvasive, can impact sleep quality and quantity in ICU patients. While some studies suggest that the mode of ventilation influences sleep, the findings have been inconsistent (Nilius et al. 2021; Bosma et al. 2007). Assisted controlled ventilation has been associated with better sleep quality compared to pressure‐supported ventilation at low‐pressure rates in patients with acute respiratory failure. However, excessive ventilation leading to hypocapnia and central sleep apnea can result in increased arousals and impaired sleep quality. Moreover, asynchrony between patients and ventilators can significantly impair sleep depth and REM sleep, contributing to increased arousals and reduced sleep efficiency. Asynchrony may account for up to 19% of sleep fragmentation in these patients (Toublanc et al. 2007; Parthasarathy and Tobin 2002).

## 5 COVID‐19

New infectious diseases, such as SARS and COVID‐19, can exacerbate anxiety, stress, and sleep disturbances due to the activation of the immune system, physical discomfort, and psychological stress (Akıncı and Melek Başar 2021). A meta‐analysis found that approximately 34% of COVID‐19 patients experience sleep disturbances, with environmental factors being the primary cause in non‐COVID‐19 patients, while physical and psychological factors are more prevalent in COVID‐19 patients (Akıncı and Melek Başar 2021; Deng et al. 2021). One study specified that poor sleep quality in COVID‐19 patients was associated with longer hospitalization, suggesting that improving sleep quality could help reduce hospital stays. Factors that increase the risk of sleep disturbances in these patients include longer hospitalization, pre‐existing mental health conditions, low lymphocyte count, and a higher neutrophil‐to‐lymphocyte ratio (Akıncı and Melek Başar 2021; van den Ende et al. 2021).

### 5.1 Comorbidities

Many comorbidities can disrupt sleep in hospitalized patients, including congestive heart failure, endocrine disorders, chronic obstructive pulmonary disease (COPD), gastroesophageal reflux disease (GERD), cardiovascular disease, pregnancy, rheumatoid arthritis, fibromyalgia, multiple sclerosis, Parkinson's disease, renal disease, obesity, and severe liver disease (Vaishnav et al. 2023; Goldstein et al. 2021; Ouellet, Beaulieu‐Bonneau 2019; Hu et al. 2024; Waheed and Kalsoom 2024; Busebee et al. 2024; Yun et al. 2024; McNicholas 1993). Parkinson's disease and Alzheimer's disease, in particular, are associated with multiple sleep disturbances that worsen as the diseases progress (Kuzmik et al. 2022; Zhang et al. 2020). Sleep disturbances can also signal underlying psychiatric disorders, such as major depressive disorder, which is characterized by specific abnormalities in sleep architecture (Breslau et al. 1996).

Obesity is another significant contributor to sleep disturbances, affecting sleep through various mechanisms. Excess visceral adipose tissue secretes inflammatory cytokines, which can disrupt the sleep‐wake cycle. A common obesity‐related sleep disorder is OSA, which increases the risk of numerous chronic conditions. Recent research also suggests that low physical activity and being overweight or obese are significant factors that disrupt sleep in hospitalized patients (Wang et al. 2022; Lima et al. 2022).

Moreover, underlying sleep disorders such as sleep‐disordered breathing, restless leg syndrome, and periodic limb movement disorder can contribute to sleep disturbances in hospitalized patients, with hospitalization often exacerbating these conditions. For example, OSA may worsen due to the supine position and the use of sedating medications during hospitalization (Oksenberg and Silverberg 2009).

*Mrs. Z's untreated restless legs syndrome and chronic insomnia likely worsened her sleep problems during hospitalization, compounding the negative effects of environmental and care‐related factors*.

**To address these underlying conditions**:

**Table:** 

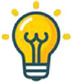	• **Medication review**: Consult with a clinical pharmacist to review her medications and identify any potential culprits for sleep disturbances. • **Non‐pharmacological interventions**: Encourage relaxation techniques, such as deep breathing and meditation, to help manage insomnia. • **Specialized treatment**: Refer her to a sleep specialist for evaluation and treatment of restless legs syndrome.

### 5.2 Medication Effects

Any medication that crosses the blood‐brain barrier can potentially affect sleep quality and architecture. Many drugs commonly used in hospitalized patients, including corticosteroids, beta‐blockers, vasopressors, and sedatives, are known to disrupt sleep patterns (Table 1) (Burger et al. 2022; Stewart and Arora 2022; Kulpatcharapong et al. 2020; Nilius et al. 2021; Clements et al. 2023; Marino et al. 2021; Auckley 2023; White et al. 2023). The timing and frequency of medication administration can also interfere with sleep, particularly if drugs are given late at night or close to bedtime (Bourne and Mills 2004).

**TABLE 1:** Medication with potential effects on sleep architecture.

Drug class (examples)		Effects on sleep	Considerations
Anticonvulsants	Traditional (phenobarbital, carbamazepine, oxcarbazepine phenytoin)	Decreasing sleep onset latency Increasing TST Increasing stage N1 and decreasing stage N3 (phenytoin) Increasing stage N3 (carbamazepine) Decreasing REM (phenobarbital)	When initiating treatment with older antiseizure drugs for sleep promotion, rapid tolerance typically develops within a week. In contrast, the sleep‐related side effects of newer agents seem to show less pronounced tolerance To avoid increased seizure frequency or other withdrawal symptoms, it is recommended to gradually taper these medications over a week or more when discontinuing their use
	Newer agents (levetiracetam)	Improving overall sleep quality Increasing stage N3 Reducing REM sleep	
	GABA‌ enhancers (gabapentin, tiagabine, pregabalin)	Decreasing the number of awakenings Decreasing sleep onset latency Increasing TST	
Antidepressants	Tertiary TCAs (doxepin, amitriptyline, trimipramine)	Decreasing sleep onset latency Decreasing wakefulness after sleep onset Suppressing REM sleep	Schedule the medication dosing to minimize disruptions to night‐time sleep Gradually taper the dose or change to an alternative medication to prevent abrupt discontinuation and associated withdrawal symptoms Anticholinergic and sedating effects of antidepressants typically develop tolerance within a week or two, while REM‐suppressing effects may persist Abrupt discontinuation of REM sleep‐suppressing antidepressants can lead to REM sleep rebound and sleep disturbance
	Secondary TCAs (nortriptyline, desimipramine)	Suppressing REM sleep	
	MAOIs (isocarboxazid, phenelzine, tranylcypromine)	Increasing wakefulness after sleep onset Decreasing TST Suppressing REM sleep	
	SARIs (trazodone, nefazodone)	Improving overall sleep quality Increasing stage N3 (trazodone) Decreasing stage N3 (nafazodone)	
	SSRIs (fluoxetine, paroxetine, sertraline)	Decreasing TST Increasing wakefulness after sleep onset Increasing stage N1 Suppressing REM sleep	
Analgesics ^a^	Opioids (codeine, morphine)	Increasing wakefulness after sleep onset (with chronic use) Decreasing stage N3	Long‐acting opioids tend to increase daytime sleepiness Rapid tolerance to this side effect typically develops within a few days
	Antipyretic analgesics (aspirin, acetaminophen, NSAIDs)	Increasing wakefulness after sleep onset (NSAIDs)	Over‐the‐counter sleeping aids and cold remedies often contain analgesics, such as acetaminophen combined with antihistamines
Atypical antipsychotics (quetiapine, ziprasidone, olanzapine, and clozapine)		Increasing TST Decreasing sleep onset latency Suppressing REM sleep Increasing stage N3	Gradually taper the dose or switch to a different medication Due to their prolonged half‐lives, atypical antipsychotic medications may cause daytime sedation
α adrenergic agonists	Clonidine	Decreasing TST in hypertensive patients Increasing TST in healthy individuals Increasing the number of shifts to stage N1 sleep or wakefulness Suppressing REM sleep	Clonidine increases daytime sleepiness Schedule the medication dosing to avoid interference with nighttime sleep
	Dexmedetomidine	Decreasing sleep onset latency Increasing stage N2 Decreasing/Increasing stage N3 Suppressing REM sleep	
Beta‐blockers	Lipophilic (carvedilol, propranolol, metoprolol, pindolol)	Increasing the number and time of awakening after sleep onset Suppressing REM sleep Associated with insomnia, nightmare, daytime sleepiness, and hallucination	Schedule the medication dosing to avoid interference with nighttime sleep
	Hydrophilic (atenolol, sotalol)	Suppressing REM sleep	
Benzodiazepines		Improving overall sleep quality Reducing sleep onset latency Reducing the amount of stage N1 sleep Increasing spindle activity during stage N2 sleep and increasing N2 percent Having differing effects on stage N3 sleep, with uncertain clinical significance Modestly reducing REM sleep, particularly at higher than indicated doses	Longer‐acting agents also decrease wakefulness after sleep onset and increase total sleep time Discontinuation of these medications may lead to rebound insomnia These medications can impair cognition, mobility, and increase the risk of falls in older adult
Non‐benzodiazepine benzodiazepine receptor agonists (zolpidem, zaleplon, eszopiclone)			
Corticosteroids		Increasing the number of awakening after sleep onset Suppressing REM sleep	The use of inhaled glucocorticoids in most patients does not seem to result in the same detrimental effects on sleep as other forms of glucocorticoids
Melatonin and melatonin receptor agonists (ramelteon, tasimelteon)		Reducing sleep onset latency Increasing sleep duration (tasimelteon) Reducing the amount of stage N1 (remelteon)	Safe and well tolerated with no evidence for withdrawal effects
Orexin receptor antagonists (suvorexant, lemborexant, and daridorexant)		Improving sleep onset and maintenance of sleep Reducing REM sleep latency	No evidence for rebound or withdrawal effects
CNS Stimulants^b^ (amphetamine, dextroamphetamine, modafinil, methylphenidate)		Increasing sleep latency to sleep onset Increasing wakefulness during the sleep Increasing N1 stage sleep Reducing N3 stage sleep Reducing REM sleep	Schedule dosing to avoid interference with night‐time sleep, consider non‐stimulant alternatives, avoid caffeine and other stimulants Rapid tolerance develops to these effects on sleep quality and staging. Upon discontinuation of the drug, there may be increased sleepiness and a rebound in REM sleep Caffeine intake disrupts sleep due to its antagonistic effects on adenosine, which regulates the homeostatic sleep drive, and by affecting circadian timing
Theophylline		Increasing sleep onset latency Decreasing sleep efficiency Decreasing TST Increasing stage N2 Decreasing stage N3	Consider administering theophyllines earlier in the day to minimize sleep disturbances Prescribe the lowest dose to control the disease and adjust the dose based on the level
Vasopressors		Decreasing TST Decreasing stage N3 Suppressing REM sleep	Frequently monitor and adjust the dose of vasopressors to find the lowest effective dose that still maintains hemodynamic stability
Propofol		Decreasing sleep onset latency Increasing TST Increasing stage N3	There is currently not enough data to establish whether propofol improves sleep quality or duration in ICU patients

Abbreviations: CNS, central nervous system; NSAIDs, non‐steroidal anti‐inflammatory drugs; REM, rapid eye movement; SARI, Serotonin‐2 antagonist/reuptake inhibitors; SSRI, selective serotonin reuptake inhibitors; TCA, three cyclic antidepressants; TST, total sleep time.

^a^ Analgesics can potentially improve sleep if pain is the underlying cause of sleep disturbance.

^b^ Certain over‐the‐counter medications contain ephedrine or pseudoephedrine, which have a chemical structure resembling amphetamine, these substances are likely to interfere with sleep.

Withdrawal reactions are common in hospitalized patients, especially those related to sedative agents used for mechanical ventilation (Hillman et al. 2023). These reactions may include insomnia, sleep disturbances, and withdrawal symptoms linked to prolonged use of high‐dose opioids and benzodiazepines. Gradual tapering of sedative doses and the use of validated sedation scoring systems can help minimize the risk of excessive dosing and withdrawal reactions (Bourne and Mills 2004).

Besides, the discontinuation of chronic medications in acutely ill patients can lead to withdrawal effects such as insomnia and nightmares, particularly with drugs that suppress REM sleep (Bourne and Mills 2004). Withdrawal from recreational substances, including alcohol, cannabis, amphetamines, cocaine, and nicotine, can also disrupt sleep in ICU patients (Li and Shoptaw 2023).

*The beta‐blocker prescribed for atrial fibrillation may have contributed to Mrs. Z's vivid dreams and restlessness, further disrupting her sleep patterns*.

**To address this**:

**Table:** 

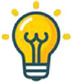	• **Medication review**: Collaborate with her cardiologist and clinical pharmacist to explore alternative medications or dosage adjustments for the beta‐blocker that may have fewer sleep‐disruptive side effects.

### 5.3 Other Factors

A significant association was also observed between sleep quality and several factors, such as the type of family, income, and education levels, suggesting that individual differences and socioeconomic factors may influence sleep quality during hospitalization (Kulpatcharapong et al. 2020).

## 6 Management of Sleep Disturbance in Hospitalized Patients

Optimizing sleep for hospitalized patients requires a comprehensive approach that involves raising awareness of sleep disturbances, implementing targeted interventions at the individual level, and creating a hospital environment conducive to restful sleep. The Society of Anesthesia and Sleep Medicine highlights the importance of systemic efforts by hospitals and healthcare providers to address sleep in hospitalized patients. Healthcare providers should be well‐informed about the various sleep disorders that can affect patients and the impact of hospitalization on sleep patterns and architecture. Recognizing the signs and symptoms of sleep disorders, along with understanding their underlying pathophysiology, is crucial for accurate diagnosis and effective treatment, ultimately improving patient outcomes and the quality of healthcare delivery.

## 7 Assessment of Sleep Disturbances in Hospitalized Patients

**FIGURE 3:**
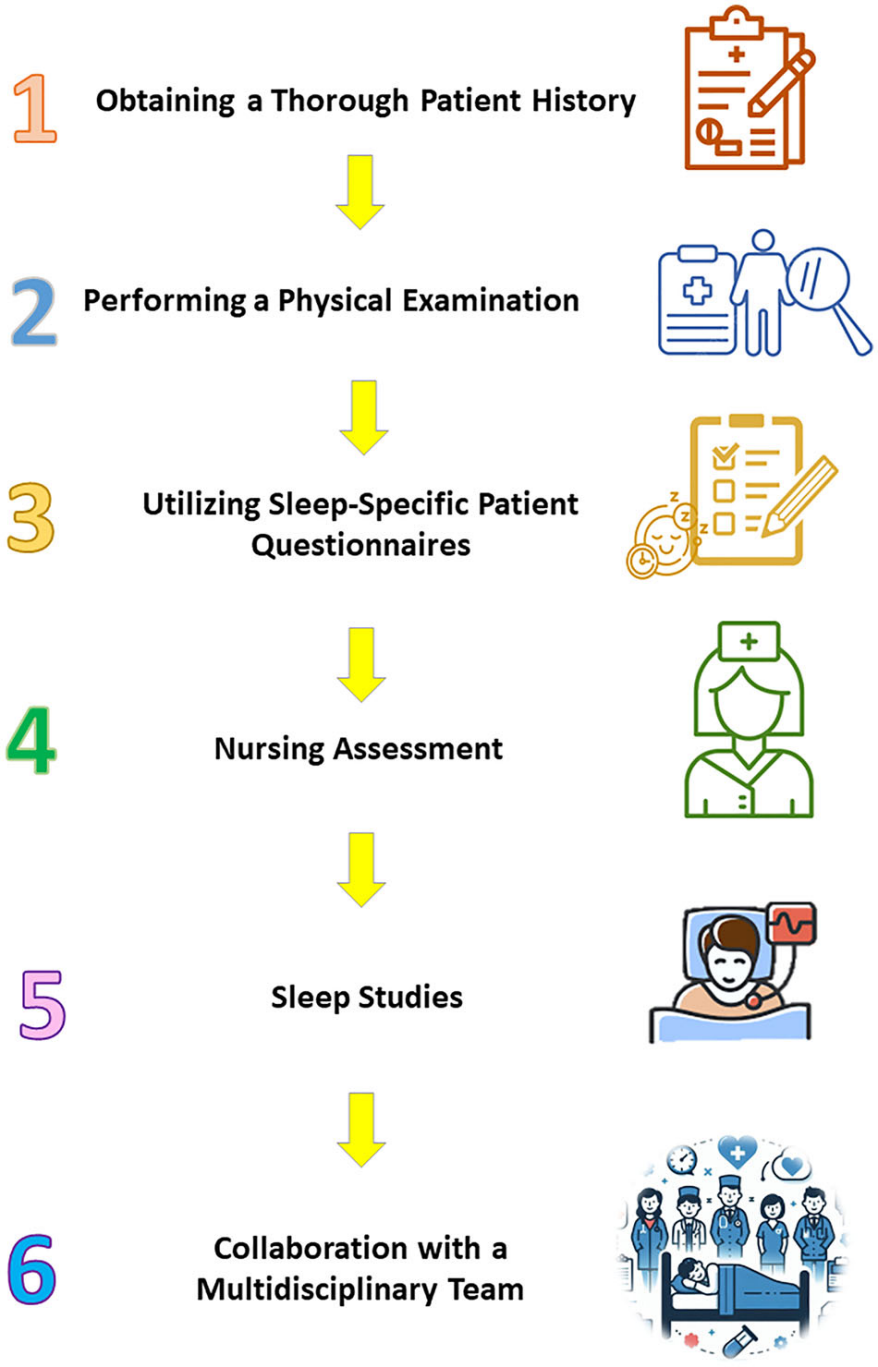
A multifaceted approach to assessing sleep disorders in hospitalized patients.

Assessing sleep disorders or disruptions in hospitalized patients is crucial for achieving optimal health outcomes. However, despite its importance, there is currently no standardized method for sleep assessment in this population. Below are some suggested steps for evaluating sleep disturbances in hospitalized patients (Figure 3):

- Obtaining a thorough patient history: A thorough patient history should include questions about the patient's sleep patterns, habits, and any pre‐existing sleep disorders prior to hospitalization. Moreover, reviewing the patient's medication history can help identify drugs that may contribute to sleep disturbances (Table 1). This information is crucial for understanding the patient's baseline sleep quality and detecting any changes that may have occurred during their hospital stay (Stewart and Arora 2022; White et al. 2023).
- Performing a physical examination: A physical examination should be conducted to assess for signs of sleep‐disordered breathing, such as snoring, obesity, or anatomical abnormalities. Vital signs, including blood pressure, heart rate, and oxygen saturation, should also be monitored, as abnormalities in these measurements may suggest sleep‐disordered breathing or other sleep‐related issues (Aeschbacher et al. 2016). Moreover, the patient can be evaluated for any signs of restlessness, leg movements, or nerve‐related conditions that could impact their sleep (Shelgikar and Chervin 2013).
- Utilizing sleep‐specific patient questionnaires: Currently, sleep assessment tools are mainly used for research purposes in acute hospital settings. Among the clinically applicable instruments for measuring inpatient sleep, the Richards–Campbell Sleep Questionnaire holds the most promise for widespread use due to its convenience and ease of administration. Although it was originally developed for use in intensive care units, it has not yet been validated for general hospital inpatients. Further research is needed to determine its suitability and effectiveness in this broader patient population (Hoey et al. 2014).
- Nursing assessment: Nurses in the ICU can monitor sleep using validated tools such as the Sleep Observation Tool (SOT) or the Echols Patient Sleep Behavior Observation Tool (PSBOT). While these tools demonstrate some validity, nursing assessments often overestimate total sleep time compared to polysomnography (PSG). For instance, ICU patients who are immobile or frequently close their eyes while awake may be inaccurately classified as asleep. Other nursing assessments, like Beecroft's questionnaire and Ibrahim's assessment, have limited reliability and criterion validity when compared to PSG (Jeffs and Darbyshire 2019; Elías 2021).
- Sleep studies: Routine use of (PSG), electroencephalography (EEG), bispectral index, or actigraphy to assess sleep in ICU patients is not recommended (Elías 2021). However, when sleep disturbances are suspected or pre‐existing sleep disorders may be worsened by hospitalization, sleep studies may be considered. These studies can help identify specific sleep disorders, such as OSA, restless leg syndrome, or periodic limb movement disorder, and guide appropriate treatment. PSG in the ICU often involves non‐sedated patients and small sample sizes, with inter‐rater reliability around 0.83. The ICU environment, however, reduces PSG reliability due to electrical artifacts and the potential disruption of patient care activities. Other drawbacks include the need for a certified sleep technician, subjectivity in scoring, discomfort from electrodes, and the high cost, all of which limit the number of critical care studies (Berry et al. 2013; Elías 2021).
- Collaboration with a multidisciplinary team: A multidisciplinary team, including physicians, nurses, sleep medicine specialists, pain management experts, psychologists, and clinical pharmacists, can play a key role in assessing and managing sleep disturbances in hospitalized patients (Clements et al. 2023).

By incorporating these assessment strategies, sleep disturbances in hospitalized patients can be better identified and addressed, ultimately improving their overall health and recovery outcomes.

### 7.1 Non‐Pharmacologic Strategies

Non‐pharmacologic strategies are essential for improving sleep quality and duration in hospitalized patients, as they typically require minimal resources and pose little risk. In older adults and patients with severe illnesses, sedative‐hypnotic medications can lead to serious adverse effects such as falls, delirium, and respiratory problems, making non‐pharmacologic approaches a safer and more advisable option for managing sleep disturbances in these vulnerable populations (Miller et al. 2019; Roehrs 2023). Among these approaches, cognitive behavioral therapy for insomnia (CBT‐I) appears to be the most promising intervention for improving sleep quality and duration in medical settings (Miller et al. 2019).

Noise reduction techniques, such as the use of earmuffs or earplugs, sound masking, installing soundproofing materials, closing doors, reducing medical equipment volume, and implementing “quiet time” protocols, have consistently shown modest improvements in subjective sleep quality and duration, even with minimal reductions in overall noise levels (Ruettgers et al. 2022).

The benefits of light therapy in hospital settings may be less significant than previously believed (Hu et al. 2015). While nocturnal light exposure is less problematic, insufficient daytime light exposure can disrupt circadian rhythms and sleep. Studies suggest that improving daytime light exposure in hospitals can enhance circadian alignment and nocturnal sleep, although the impact on total sleep time remains unclear. This approach may not be effective for all patient populations (Beswick et al. 2023; Chambe et al. 2023).

Reducing nighttime interruptions by altering hospital workflows such as delaying or minimizing vital sign checks, using passive monitoring, rescheduling medications, and postponing morning procedures like phlebotomy and X‐rays can improve sleep (Daniel et al. 2024). While the rationale for limiting nighttime interruptions is supported by evidence, further research is needed to optimize these changes and ensure safe implementation to enhance sleep consolidation, improve quality, and positively impact patient outcomes (Knauert et al. 2019).

Relaxation techniques, including music, massage, guided imagery, and aromatherapy, have shown minor improvements in subjective or nurse‐determined sleep quality and duration (Miller et al. 2019). Given the safety and low cost of these interventions, they should be considered on a case‐by‐case basis (Stewart and Arora 2022).

If insomnia is related to an underlying medical condition, treating that condition can improve sleep quality. Symptoms that hinder sleep, such as coughing or nocturnal urination, should be addressed. Cough can be treated with a suppressant, and nocturnal urination can be minimized by reducing diuretic use, particularly near bedtime. Elevating the head of the bed at a 30‐degree angle or higher can benefit patients with heart failure, COPD, GERD, and stroke (Stewart and Arora 2022). Multifaceted protocols combining environmental adjustments with prescriber and caregiver education have shown promise, but further work is needed to ensure consistent improvements in patient‐centered sleep outcomes (Stewart and Arora 2022).

## 8 Pharmacotherapy

Pharmacotherapy is commonly used to manage sleep disturbances during hospitalization. Approximately 20–30% of hospitalized patients receive sleep medications, and 10–30% continue their use after discharge (Mansour and Knauert 2022). However, there is limited data on the safety and efficacy of these agents in the hospital setting. One institutional review found that 26% of inpatient admissions were prescribed sleep aids (Gillis et al. 2014), while another study reported that 16% of older inpatients were prescribed new benzodiazepines or sedative‐hypnotics, 77% of which were deemed inappropriate (Pek et al. 2017). Although nonpharmacologic interventions are generally recommended as the first line of treatment, pharmacological options may be necessary in some cases (Mansour and Knauert 2022).

For insomnia treatment, medication selection should be individualized based on symptom severity, age, comorbidities, side effects, and drug‐drug interactions (Table 2).

**TABLE 2:** Medications used to treat sleep disturbances during hospitalization.

Drug name	Indication^a^	Dose, administration	Important drug interactions	Considerations for use in hospitalized patients
Melatonin	Insomnia Circadian rhythm disorders	1–5 mg as needed before bedtime	Antihypertensive medications (nifedipine), Alcohol, sedatives, and other CNS ‌depressants	Adverse effects are usually mild and avoidable Monitor blood pressure and heart rate in patients on nifedipine
Ramelteon^c^	Insomnia Circadian rhythm disorders	8 mg, as needed, within 30 min of bedtime Do not take with a high‐fat meal	Alcohol, sedatives, and CNS depressants CYP1A2 Inhibitors CYP3A4 inhibitors and inducers CYP2C9 Inhibitors	Use with caution in patients with liver impairment, sleep apnea, and respiratory disorders Monitor for complex sleep behaviors and withdrawal symptoms Not recommended in older adults
BZDs^C^, ^d^	Insomnia (sleep onset)	Refer to individual agent monograph	Alcohol, sedatives, opioids, and CNS depressants Use with caution in patients with liver impairment, sleep apnea, and respiratory disorders. Monitor for complex sleep behaviors and withdrawal symptoms. Not recommended in older adults.	Use with caution in patients with liver impairment, sleep apnea, respiratory disorders, substance abuse, and older adults. Monitor for withdrawal symptoms and risks of abuse, misuse, and addiction. Risks of abuse, misuse, and addiction, which can lead to overdose or death
Zolpidem^c^	Insomnia (sleep onset and maintenance)	5–10 mg before bedtime; do not administer with food	Alcohol, sedatives, and CNS depressants St. John's Wort CYP3A4 inhibitors and inducers	Use with caution in patients with liver impairment, sleep apnea, and respiratory disorders May cause complex sleep behaviors Not recommended in older adults
Trazodone^c^	Insomnia (sleep onset and maintenance) Insomnia in patients with depression	25–100mg before bedtime (max dose: 300 mg) Administer shortly after a meal or light snack Do not take within 3 h of high‐fat meals	Alcohol, sedatives, QT‐prolonging agents, serotonergic agents	Use with caution in patients with coronary artery disease, kidney impairment, liver impairment, and seizure disorders Monitor for serotonin syndrome and withdrawal symptoms
Doxepin^c^	Insomnia (sleep maintenance)	Tablets: 3–6 mg before bedtime Capsules: 10 mg before bedtime	May interact with alcohol, sedatives, and other sleep aids QT‐prolonging agents Serotonergic agents Anticholinergic agents	Use with caution in patients with heart conditions, kidney impairment, liver impairment, and seizure disorders Monitor for withdrawal symptoms due to its prolonged half‐life
Gabapentin^c^	Restless leg syndrome Insomnia	100–300 mg, 2 h before bedtime	Alcohol, sedatives, and CNS depressants	Use with caution in patients with kidney impairment, respiratory disorders, myasthenia gravis, seizure disorders, substance abuse, and heart conditions Withdraw gradually to minimize the potential of increased seizure frequency or other withdrawal symptoms
Pregabalin^c^	Restless leg syndrome Insomnia	50 to 75 mg once daily, 1 to 3 h before bedtime	Alcohol, sedatives, and CNS depressants	Use with caution in patients with kidney impairment, respiratory disorders, seizure disorders, substance abuse, and heart conditions Withdraw gradually to minimize the potential of increased seizure frequency or other withdrawal symptoms
Quetiapine^c^	Insomnia	25–100 mg before bedtime Administer without food or with a light meal (≤300 calories)	Alcohol, sedatives, and CNS depressants QT‐prolonging agents Serotonergic agents Anticholinergic agents	Use with caution in patients with heart conditions, kidney impairment, and seizure disorders May cause orthostatic hypotension, anticholinergic effects, movement disorder, and QT prolongation Withdrawal symptoms are likely with abrupt discontinuation

Abbreviations: BZD, benzodiazepines; CNS, central nervous system.

^a^ Indications for use in sleep‐related problems.

^b^ Immediate release melatonin preparations should be given 30 to 60 min before bedtime, and sustained release preparations should be given 1–2 h before bedtime.

^C^ Limit long‐term use (>4 weeks) to cases for which nonpharmacologic treatments are not available or not effective and benefits are felt to outweigh risks.

^d^ Benzodiazepines approved for use in insomnia includes estazolam, flurazepam, quazepam, temazepam, triazolam.

### 8.1 Melatonin and Melatonin Agonists

Melatonin is often considered the preferred first‐line treatment for sleep disturbances due to its mild side effect profile, low potential for drug‐drug interactions, and ability to help regulate circadian rhythms (Roehrs 2023; Haider et al. 2024). Studies have shown improvements in sleep quality with melatonin use (Foley and Steel 2019; Salahub et al. 2023). Although small trials evaluating exogenous melatonin in ICU settings have yielded conflicting results, a systematic review of randomized controlled trials found insufficient evidence to determine its impact on sleep, mortality, or delirium in ICU patients (Lewis et al. 2018). A meta‐analysis of two randomized controlled trials in hospitalized patients, however, found that melatonin and ramelteon were associated with improved sleep quality, increased nighttime sleep duration, and fewer nighttime awakenings (Khaing and Nair 2021). A recent meta‐analysis concluded that there is currently insufficient evidence to assess the effect of melatonin and ramelteon on delirium incidence in ICU patients, noting that the available data are of low quality and calling for further studies to confirm these findings (Aiello et al. 2023).

### 8.2 Benzodiazepines and Non‐Benzodiazepine Benzodiazepine Receptor Agonists

Benzodiazepines (BZDs) are the most commonly prescribed hypnotics in hospitals, with five BZDs approved for the treatment of insomnia (Table 2). However, caution is warranted when using BZDs in the inpatient setting, particularly in older adults, due to their association with significant adverse effects, such as residual daytime sedation, anterograde amnesia, and respiratory depression. BZDs are contraindicated in patients with COPD, OSA, or a history of drug abuse. Long‐acting BZDs, such as flurazepam and quazepam, should be avoided as they carry the highest risk of daytime sedation. Triazolam, with a short half‐life, is not recommended for long‐term use due to its potential for withdrawal, dependence, and tolerance. Estazolam and temazepam are the most appropriate options in the hospital setting, given their moderate duration and quick‐to‐moderate onset of action. Lorazepam, while not approved as a hypnotic, is sometimes used as a sleep aid in hospitals due to its similar pharmacokinetics to temazepam (Frighetto et al. 2004).

Although BZDs can decrease sleep latency in hospitalized patients, their effect on total sleep time and overall sleep quality is limited (White et al. 2023). A systematic review has shown that sedative‐hypnotics, including BZDs, are similarly effective to placebo or no treatment, and also to each other (Kanji et al. 2016). Non‐benzodiazepine BZD receptor agonists (Z‐drugs), such as zolpidem, eszopiclone, and zaleplon, are commonly used in the inpatient setting and have shown effectiveness in the outpatient setting. However, their use in hospitalized patients has been linked to cognitive dysfunction, delirium, and falls (Kosto et al. 2023; Heinemann et al. 2020).

In older adults (age ≥ 65), the use of sedative‐hypnotics has been associated with an increased risk of falls and hip fractures, and these medications are generally avoided in this population (Kolla et al. 2013, Finkle et al. 2011). They are listed as potentially inappropriate medications in the American Geriatrics Society Beers Criteria (American Geriatrics Society 2023). Long‐term use of BZDs in older adults is also linked to an increased risk of cognitive decline, dementia, and delirium (Liu et al. 2020). Z‐drugs carry similar risks and are recommended for short‐term use only and not in combination with BZDs (American Geriatrics Society 2023). Furthermore, new benzodiazepine prescriptions can lead to prolonged use, with withdrawal symptoms such as insomnia, anxiety, and tremors occurring when discontinuing these medications after long‐term use (Bell et al. 2007; Edinoff et al. 2021).

## 9 Antihistamines

Antihistamines, such as diphenhydramine and doxylamine, have a long history of use and are generally considered safe. However, their efficacy as sleep aids is limited, and their side effects may be more pronounced in the inpatient setting. Antihistamines should not be used long‐term, as tolerance can develop in less than a week (Young et al. 2009). First‐generation antihistamines, including diphenhydramine, are listed as potentially inappropriate medications in the American Geriatrics Society Beers Criteria due to the risk of anticholinergic effects such as confusion, sedation, and delirium. They are not recommended as hypnotics (American Geriatrics Society 2023).

A large prospective cohort study found that elderly hospitalized patients receiving diphenhydramine had a 70% increased risk of cognitive decline compared to those not receiving the medication. The treatment group also experienced more behavioral disturbances and a higher incidence of urinary catheter placement, which may be indicative of anticholinergic effects such as delirium, agitation, and urinary retention (Adinoff 2002). Given its anticholinergic properties, diphenhydramine is not recommended for use as a hypnotic in hospitalized patients, particularly those who are prone to such side effects (American Geriatrics Society 2023).

## 10 Antidepressants

Antidepressants, including trazodone, mirtazapine, and tricyclic antidepressants (TCAs), have been used to treat insomnia. However, TCAs are generally not recommended, especially in older adults, due to their anticholinergic effects and adverse cardiovascular risks. Mirtazapine may be beneficial for patients with insomnia, depression, and anorexia, such as those with cancer or AIDS, as it can stimulate appetite at low doses (Young et al. 2009). Trazodone, a serotonin modulator with sedative properties, is often better tolerated than benzodiazepines but should be used with caution in patients at risk for orthostatic hypotension or arrhythmias, including both atrial and ventricular types.

Low‐dose doxepin has shown promise for sleep maintenance insomnia in the outpatient setting, but data on its use in hospitalized patients is limited. Other classes of antidepressants have not been well studied in the inpatient setting, and they carry potential risks, particularly related to side effects and interactions with other medications (Marino et al. 2021).

### 10.1 Gabapentin and Pregabalin

Gabapentin has shown consistent efficacy in managing sleep disturbances in patients with medical illnesses. However, it carries a relatively high risk of treatment discontinuation and withdrawal symptoms. Adverse events are more common during the dose‐increasing phase, particularly at higher doses. To minimize these risks, a slower dose escalation and lower maintenance doses of gabapentin may help reduce the likelihood of adverse events (Liu et al. 2017).

## 11 Antipsychotics

Atypical antipsychotics, including quetiapine, risperidone, and olanzapine, may be used to treat insomnia in patients experiencing acute psychotic or manic episodes. However, caution is advised when prescribing these medications due to their potential side effects. Given the lack of high‐quality randomized clinical trials supporting their efficacy and safety for insomnia, the off‐label use of quetiapine, especially in elderly hospitalized patients, should be limited (Lee et al. 2016).

### 11.1 Patients With Chronic Sleep Disorders

Patients with chronic insomnia who are receiving long‐term pharmacotherapy should be reassessed during hospitalization to evaluate the continued need for treatment. If ongoing therapy is required, key considerations include potential drug–drug interactions, new organ dysfunction, and altered susceptibility to side effects due to the patient's current illness (Auckley 2023).

**Successful Management**

**Table:** 

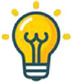	• *Non‐pharmacological interventions, such as earplugs, eye masks, relocation to a quieter room, reduced nighttime interruptions, and adjusted bed positioning, significantly improved Mrs. Z's sleep and reduced her fatigue*. • *Pharmacological treatments were tailored to address Mrs. Z's specific needs. Switching her beta‐blocker from metoprolol tartrate to bisoprolol relieved vivid dreams and restlessness. Melatonin was prescribed to regulate her sleep‐wake cycle, and gabapentin was introduced to manage her restless legs syndrome. These targeted therapies, combined with non‐pharmacological measures, helped to improve her overall sleep quality*.

## 12 Conclusion

Managing sleep disturbances in hospitalized patients requires a multifaceted approach that involves understanding sleep disorders, the impact of hospitalization on sleep patterns, and effective assessment strategies. Proper diagnosis and treatment of these sleep disturbances are essential for improving patient outcomes and enhancing overall healthcare delivery.

## Author Contributions

**Ghazal Roostaei**: writing–review and editing, writing–original draft, data curation. **Niloofar Khoshnam Rad**: methodology, data curation, writing–review and editing, writing–original draft, conceptualization, investigation, validation, supervision. **Besharat Rahimi**: supervision, conceptualization. **Alireza Asgari**: data curation, writing–original draft. **Shima Mosalanejad**: data curation, writing–original draft. **Hossein Kazemizadeh**: validation, investigation. **Maryam Edalatifard**: supervision. **Hamidreza Abtahi**: conceptualization, writing–review and editing, methodology.

### Peer Review

The peer review history for this article is available at https://publons.com/publon/10.1002/brb3.70282.

## Data Availability

Data sharing is not applicable to this article as no new data were created or analyzed in this study.

## References

[brb370282-bib-0001] Adinoff, A. 2002. “Cognitive and Other Adverse Effects of Diphenhydramine Use in Hospitalized Older Patients.” Pediatrics 110, no. Supplement_2: 442–443. 10.1542/peds.110.s2.442a.

[brb370282-bib-0002] Aeschbacher, S. , M. Bossard , T. Schoen , et al. 2016. “Heart Rate Variability and Sleep‐Related Breathing Disorders in the General Population.” American Journal of Cardiology 118, no. 6: 912–917. 10.1016/j.amjcard.2016.06.032. 27553103

[brb370282-bib-0003] Aiello, G. , M. Cuocina , and L. La Via , et al. 2023. “Melatonin or Ramelteon for Delirium Prevention in the Intensive Care Unit: A Systematic Review and Meta‐Analysis of Randomized Controlled Trials.” Journal of Clinical Medicine 12, no. 2: 435. 10.3390/jcm12020435. 36675363 PMC9863078

[brb370282-bib-0004] Akıncı, T. , and H. Melek Başar . 2021. “Relationship Between Sleep Quality and the Psychological Status of Patients Hospitalised With COVID‐19.” Sleep Medicine 80: 167–170. 10.1016/j.sleep.2021.01.034. 33601228 PMC7842153

[brb370282-bib-0005] Almeneessier, A. S. , B. N. Alamri , F. R. Alzahrani , and M. M. Sharif . 2018. “Pandi‐perumal SR, Bahammam AS. Insomnia in Primary Care Settings: Still Overlooked and Undertreated?” Journal of Natural Medicines 1, no. 2: 64–68. 10.4103/JNSM.JNSM_30_18.

[brb370282-bib-0006] American Geriatrics Society . 2023. “Updated AGS Beers Criteria® for Potentially Inappropriate Medication Use in Older Adults.” Journal of the American Geriatrics Society 71, no. 7: 2052–2081. 10.1111/jgs.18372. 37139824 PMC12478568

[brb370282-bib-0007] Auckley, D. H. 2023. Poor Sleep and Insomnia in Hospitalized Adults. In Uptodate. Wolters Kluwer, Netherlands.

[brb370282-bib-0008] Bell, C. M. , H. D. Fischer , S. S. Gill , et al. 2007. “Initiation of Benzodiazepines in the Elderly After Hospitalization.” Journal of General Internal Medicine 22, no. 7: 1024–1029. 10.1007/s11606-007-0194-4. 17453266 PMC2330138

[brb370282-bib-0009] Berry, R. B. , R. Brooks , C. E. Gamaldo , S. M. Harding , C. L. Marcus , and B. V. Vaughn . 2013. “The AASM Manual for the Scoring of Sleep and Associated Events.” American Academy of Sleep Medicine 53, no. 9: 1689–1699.

[brb370282-bib-0010] Besedovsky, L. , T. Lange , and M. Haack . 2019. “The Sleep‐Immune Crosstalk in Health and Disease.” Physiological Reviews 99, no. 3: 1325–1380. 10.1152/physrev.00010.2018. 30920354 PMC6689741

[brb370282-bib-0011] Beswick, A. D. , V. Wylde , W. Bertram , and K. Whale . 2023. “The Effectiveness of Non‐Pharmacological Sleep Interventions for Improving Inpatient Sleep in Hospital: A Systematic Review and Meta‐Analysis.” Sleep Medicine 107: 243–267. 10.1016/j.sleep.2023.05.004. 37257367

[brb370282-bib-0012] Blackwell, T. L. , S. C. Robinson , N. Thompson , et al. 2024. “Objective and Subjective Sleep Characteristics in Hospitalized Older Adults and Their Associations to Hospital Outcomes.” Frontiers in Sleep 3: 1346642. 10.3389/frsle.2024.1346642. PMC12713864 41424500

[brb370282-bib-0013] Bosma, K. , G. Ferreyra , C. Ambrogio , et al. 2007. “Patient‐Ventilator Interaction and Sleep in Mechanically Ventilated Patients: Pressure Support versus Proportional Assist Ventilation.” Critical Care Medicine 35, no. 4: 1048–1054. 10.1097/01.CCM.0000260055.64235.7C. 17334259

[brb370282-bib-0014] Bourne, R. S. , and G. H. Mills . 2004. “Sleep Disruption in Critically Ill Patients—Pharmacological Considerations.” Anaesthesia 59, no. 4: 374–384. 10.1111/j.1365-2044.2004.03664.x. 15023109

[brb370282-bib-0015] Breslau, N. , T. Roth , L. Rosenthal , and P. Andreski . 1996. “Sleep Disturbance and Psychiatric Disorders: A Longitudinal Epidemiological Study of Young Adults.” Biological Psychiatry 39, no. 6: 411–418. 10.1016/0006-3223(95)00188-3. 8679786

[brb370282-bib-0016] Burger, P. , E. S. Van den Ende , W. Lukman , et al. 2022. “Sleep in Hospitalized Pediatric and Adult Patients—A Systematic Review and Meta‐analysis.” Sleep Medicine: X 4: 100059. 10.1016/j.sleepx.2022.100059. 36406659 PMC9672415

[brb370282-bib-0017] Busebee, B. , K. D. Watt , K. Dupuy‐McCauley , and H. DuBrock . 2024. “Sleep Disturbances in Chronic Liver Disease.” Liver Transplant 30, no. 10: 1058–1071. 10.1097/LVT.0000000000000369 38535627

[brb370282-bib-0018] Catalá, P. , L. Gutiérrez , C. Écija , and C. Peñacoba . 2023. “Pathological Cycle Between Pain, Insomnia, and Anxiety in Women With Fibromyalgia and Its Association With Disease Impact.” Biomedicines 11, no. 1: 148. 10.3390/biomedicines11010148. 36672659 PMC9855835

[brb370282-bib-0019] Chambe, J. , E. Reynaud , J. Maruani , E. Fraih , P. A. Geoffroy , and P. Bourgin . 2023. “Light Therapy in Insomnia Disorder: A Systematic Review and Meta‐analysis.” Journal of Sleep Research 32, no. 6: e13895. 10.1111/jsr.13895. 37002704

[brb370282-bib-0020] Chang, V. A. , R. L. Owens , and J. N. LaBuzetta . 2020. “Impact of Sleep Deprivation in the Neurological Intensive Care Unit: A Narrative Review.” Neurocritical Care 32, no. 2: 596–608. 10.1007/s12028-019-00795-4. 31410770 PMC7222162

[brb370282-bib-0021] Cicolin, A. , and L. Ferini‐Strambi . 2023. “Focus on Multidisciplinary Aspects of Sleep Medicine.” Brain Sciences 13, no. 9: 1327. 10.3390/brainsci13091327. 37759928 PMC10526939

[brb370282-bib-0022] Clements, J. , E. Bowman , R. Tolhurst , et al. 2023. “The Role of the Clinical Pharmacist in the Respiratory or Sleep Multidisciplinary Team.” Breathe 19, no. 4: 230123. 10.1183/20734735.0123-2023. 38125801 PMC10729827

[brb370282-bib-0023] Daniel, L. C. , K. L. Venella , K. Woodard , et al. 2024. “Can Extending Time Between Vital Sign Checks Improve Sleep in Hematopoietic Stem Cell Transplant Patients? Testing Feasibility, Acceptability, and Preliminary Efficacy.” Pediatric Blood & Cancer 71, no. 4: e30832. 10.1002/pbc.30832. 38197636

[brb370282-bib-0024] Deng, J. , F. Zhou , W. Hou , et al. 2021. “The Prevalence of Depression, Anxiety, and Sleep Disturbances in COVID‐19 Patients: A Meta‐Analysis.” Annals of the New York Academy of Sciences 1486, no. 1: 90–111. 10.1111/nyas.14506. 33009668 PMC7675607

[brb370282-bib-0025] DePietro, R. H. , K. L. Knutson , L. Spampinato , et al. 2017. “Association Between Inpatient Sleep Loss and Hyperglycemia of Hospitalization.” Diabetes Care 40, no. 2: 188–193. 10.2337/dc16-1683. 27903614 PMC5250691

[brb370282-bib-0026] Edinoff, A. N. , C. A. Nix , J. Hollier , et al. 2021. “Benzodiazepines: Uses, Dangers, and Clinical Considerations.” Neurology International 13, no. 4: 594–607. 10.3390/neurolint13040059. 34842811 PMC8629021

[brb370282-bib-0027] Elías, M. N. 2021. “Assessment and Monitoring of Sleep in the Intensive Care Unit.” Critical Care Nursing Clinics of North America 33, no. 2: 109–119. 10.1016/j.cnc.2021.01.008. 34023079 PMC8144540

[brb370282-bib-0028] Finkle, W. D. , J. S. Der , S. Greenland , et al. 2011. “Risk of Fractures Requiring Hospitalization After an Initial Prescription for Zolpidem, Alprazolam, Lorazepam, or Diazepam in Older Adults.” Journal of the American Geriatrics Society 59, no. 10: 1883–1890. 10.1111/j.1532-5415.2011.03591.x. 22091502

[brb370282-bib-0029] Fishbein, A. B. , K. L. Knutson , and P. C. Zee . 2021. “Circadian Disruption and Human Health.” Journal of Clinical Investigation 131, no. 19: e148286. 10.1172/JCI148286. 34596053 PMC8483747

[brb370282-bib-0030] Foley, H. M. , and A. E. Steel . 2019. “Adverse Events Associated With Oral Administration of Melatonin: A Critical Systematic Review of Clinical Evidence.” Complementary Therapies in Medicine 42: 65–81. 10.1016/j.ctim.2018.11.003. 30670284

[brb370282-bib-0031] Frighetto, L. , C. Marra , S. Bandali , K. Wilbur , T. Naumann , and P. Jewesson . 2004. “An Assessment of Quality of Sleep and the Use Drugs With Sedating Properties in Hospitalized Adult Patients.” Health and Quality of Life Outcomes 2: 17. 10.1186/1477-7525-2-17. 15040803 PMC521202

[brb370282-bib-0032] Gabor, J. Y. , A. B. Cooper , S. A. Crombach , et al. 2003. “Contribution of the Intensive Care Unit Environment to Sleep Disruption in Mechanically Ventilated Patients and Healthy Subjects.” American Journal of Respiratory and Critical Care Medicine 167, no. 5: 708–715. 10.1164/rccm.2201090. 12598213

[brb370282-bib-0033] Gillis, C. M. , J. O. Poyant , J. R. Degrado , L. Ye , K. E. Anger , and R. L. Owens . 2014. “Inpatient Pharmacological Sleep Aid Utilization Is Common at a Tertiary Medical Center.” Journal of Hospital Medicine 9, no. 10: 652–657. 25130534 10.1002/jhm.2246

[brb370282-bib-0034] Goldstein, C. A. , M. Rizvydeen , and D. A. Conroy , et al. 2021. “The Prevalence and Impact of Pre‐Existing Sleep Disorder Diagnoses and Objective Sleep Parameters in Patients Hospitalized for COVID‐19.” Journal of Clinical Sleep Medicine 17, no. 5: 1039–1050. 10.5664/jcsm.9132. 33560208 PMC8320480

[brb370282-bib-0035] Haider, M. A. , K. W. Lawrence , T. Christensen , R. Schwarzkopf , W. Macaulay , and J. C. Rozell . 2024. “Does Melatonin Improve Sleep Following Primary Total Knee Arthroplasty? A Randomized, Double‐Blind, Placebo‐Controlled Trial.” Journal of Arthroplasty 39, no. 8: S154–S160. 10.1016/j.arth.2024.02.031. 38401621

[brb370282-bib-0036] Heinemann, S. , J. Brockmöller , Y. Hagmayer , and W. Himmel . 2020. “Why Z‐Drugs are Used Even if Doctors and Nurses Feel Unable to Judge Their Benefits and Risks—A Hospital Survey.” European Journal of Clinical Pharmacology 76, no. 2: 285–290. 10.1007/s00228-019-02783-1. 31732756

[brb370282-bib-0037] Hillman, D. R. 2021. “Sleep Loss in the Hospitalized Patient and Its Influence on Recovery From Illness and Operation.” Anesthesia and Analgesia 132, no. 5: 1314–1320. 10.1213/ANE.0000000000005323. 33857973

[brb370282-bib-0038] Hillman, D. R. , M. Carlucci , J. G. Charchaflieh , et al. 2023. “Society of Anesthesia and Sleep Medicine Position Paper on Patient Sleep During Hospitalization.” Anesthesia and Analgesia 136, no. 4: 814–824. 10.1213/ANE.0000000000006395. 36745563

[brb370282-bib-0039] Hoey, L. M. , P. Fulbrook , and J. A. Douglas . 2014. “Sleep Assessment of Hospitalised Patients: A Literature Review.” International Journal of Nursing Studies 51, no. 9: 1281–1288. 10.1016/j.ijnurstu.2014.02.001. 24636444

[brb370282-bib-0040] Hou, H. , S. Wu , Y. Qiu , F. Song , and L. Deng . 2022. “The Effects of Morning/Afternoon Surgeries on the Early Postoperative Sleep Quality of Patients Undergoing General Anesthesia.” BMC Anesthesiology 22, no. 1: 286. https://bmcanesthesiol.biomedcentral.com/%0Ahttp://ovidsp.ovid.com/ovidweb.cgi?T=JS&PAGE=reference&D=emexb&NEWS=N&AN=2019058438. 36088298 10.1186/s12871-022-01828-w PMC9463857

[brb370282-bib-0041] Hu, K. Y. , P. H. Tseng , W. C. Hsu , et al. 2024. “Association of Self‐Reported and Objective Sleep Disturbance With the Spectrum of Gastroesophageal Reflux Disease.” Journal of Clinical Sleep Medicine 20, no. 6: 911–920. 10.5664/jcsm.11028. 38300823 PMC11145051

[brb370282-bib-0042] Hu, R. F. , X. Y. Jiang , K. M. Hegadoren , and Y. H. Zhang . 2015. “Effects of Earplugs and Eye Masks Combined With Relaxing Music on Sleep, Melatonin and Cortisol Levels in ICU Patients: A Randomized Controlled Trial.” Critical Care (London, England) 19, no. 1: 115. 10.1186/s13054-015-0855-3. 25881268 PMC4391192

[brb370282-bib-0043] Isaia, G. , L. Corsinovi , M. Bo , et al. 2011. “Insomnia Among Hospitalized Elderly Patients: Prevalence, Clinical Characteristics and Risk Factors.” Archives of Gerontology and Geriatrics 52, no. 2: 133–137. 10.1016/j.archger.2010.03.001. 20338647

[brb370282-bib-0044] Jeffs, E. L. , and J. L. Darbyshire . 2019. “Measuring Sleep in the Intensive Care Unit: A Critical Appraisal of the Use of Subjective Methods.” Journal of Intensive Care Medicine 34, no. 9: 751–760. 10.1177/0885066617712197. 28631532

[brb370282-bib-0045] Kamdar, B. B. , D. M. Needham , and N. A. Collop . 2012. “Sleep Deprivation in Critical Illness: Its Role in Physical and Psychological Recovery.” Journal of Intensive Care Medicine 27, no. 2: 97–111. 10.1177/0885066610394322. 21220271 PMC3299928

[brb370282-bib-0046] Kanji, S. , A. Mera , B. Hutton , et al. 2016. “Pharmacological Interventions to Improve Sleep in Hospitalised Adults: A Systematic Review.” BMJ Open 6, no. 7: e012108. 10.1136/bmjopen-2016-012108. PMC4986185 27473952

[brb370282-bib-0047] Khaing, K. , and B. R. Nair . 2021. “Melatonin for Delirium Prevention in Hospitalized Patients: A Systematic Review and Meta‐Analysis.” Journal of Psychiatric Research 133: 181–190. 10.1016/j.jpsychires.2020.12.020. 33348252

[brb370282-bib-0048] Knauert, M. P. , M. Pisani , N. Redeker , et al. 2019. “Pilot Study: An Intensive Care Unit Sleep Promotion Protocol.” BMJ Open Respir Research 6, no. 1: e000411. 10.1136/bmjresp-2019-000411. PMC6561389 31258916

[brb370282-bib-0049] Kolla, B. P. , J. K. Lovely , M. P. Mansukhani , and T. I. Morgenthaler . 2013. “Zolpidem is Independently Associated With Increased Risk of Inpatient Falls.” Journal of Hospital Medicine 8, no. 1: 1–6. 10.1002/jhm.1985. 23165956

[brb370282-bib-0050] Koshy, K. , M. Gibney , D. M. O'Driscoll , R. P. Ogeil , and A. C. Young . 2024. “Factors Affecting Sleep Quality in Hospitalised Patients.” Sleep and Breathing 28, no. 6: 2737–2740. 10.1007/s11325-024-03144-8. 39243288 PMC11568048

[brb370282-bib-0051] Kosto, A. , D. Lev , N. Reiss , T. Meged‐Book , and Y. Press . 2023. “Discontinuation of Benzodiazepines and Z‐drugs in Hospitalised Population at the Age of 60 and Above. An Open‐Label Randomized Controlled Trial.” International Journal of Geriatric Psychiatry 38, no. 10: e6012. 10.1002/gps.6012. 37807766

[brb370282-bib-0052] Kulpatcharapong, S. , P. Chewcharat , K. Ruxrungtham , et al. 2020. “Sleep Quality of Hospitalized Patients, Contributing Factors, and Prevalence of Associated Disorders.” Sleep Disorders 2020: 8518396. 10.1155/2020/8518396. 32308998 PMC7157800

[brb370282-bib-0053] Kuzmik, A. , M. Boltz , R. Belue , J. E. Galvin , R. Arendacs , and B. Resnick . 2022. “Factors Associated With Sleep Quality in Hospitalized Persons With Dementia.” Alzheimer Disease and Associated Disorders 36, no. 3: 253–258. 10.1097/WAD.0000000000000502. 36001764 PMC9426998

[brb370282-bib-0054] Lee, T. C. , P. Desforges , J. Murray , R. R. Saleh , and E. G. McDonald . 2016. “Off‐Label Use of Quetiapine in Medical Inpatients and Postdischarge.” JAMA Internal Medicine 176, no. 9: 1390–1391. 10.1001/jamainternmed.2016.3309. 27400391

[brb370282-bib-0055] Lewis, S. R. , M. W. Pritchard , O. J. Schofield‐Robinson , P. Alderson , and A. F. Smith . 2018. “Melatonin for the Promotion of Sleep in Adults in the Intensive Care Unit.” Cochrane Database of Systematic Reviews 5, no. 5: CD012455. 10.1002/14651858.CD012455.pub2. 29746721 PMC6353085

[brb370282-bib-0056] Li, M. J. , and S. J. Shoptaw . 2023. “Clinical Management of Psychostimulant Withdrawal: Review of the Evidence.” Addiction 118, no. 4: 750–762. 10.1111/add.16093. 36401591 PMC10069411

[brb370282-bib-0057] Lima, R. O. , M. B. P. Landim , França de , et al. 2022. “Subjective Sleep Pattern in Hospitalized Patients.” Sleep Science 15: 120–127. 10.5935/1984-0063.20220010. 35273757 PMC8889974

[brb370282-bib-0058] Liu, G. J. , M. R. Karim , L. L. Xu , et al. 2017. “Efficacy and Tolerability of gabapentin in Adults With Sleep Disturbance in Medical Illness: A Systematic Review and Meta‐Analysis.” Frontiers in Neurology 8: 316. 10.3389/fneur.2017.00316. 28769860 PMC5510619

[brb370282-bib-0059] Liu, L. , L. Jia , P. Jian , et al. 2020. “The Effects of Benzodiazepine Use and Abuse on Cognition in the Elders: A Systematic Review and Meta‐Analysis of Comparative Studies.” Frontiers in Psychiatry 11: 00755. 10.3389/fpsyt.2020.00755. 33093832 PMC7527532

[brb370282-bib-0060] Locihová, H. , K. Axmann , P. Štěpánová , B. Břegová , R. Zittová , and J. Hrušková . 2023. “Analysis of Nighttime Sleep and the Impact of Selected Predictors (Pain and Delirium) on Its Quality in Hospitalized Patients Over Sixty Years of Age.” Journal of Turkish Sleep Medicine 10, no. 3: 221–228. 10.4274/tjsm.galenos.2023.03016.

[brb370282-bib-0061] Mansour, W. , and M. Knauert . 2022. “Adding Insult to Injury: Sleep Deficiency in Hospitalized Patients.” Clinics in Chest Medicine 43, no. 2: 287–303. 10.1016/j.ccm.2022.02.009. 35659026 PMC9177053

[brb370282-bib-0062] Marconi, E. , S. Bracci , L. Dinapoli , et al. 2024. “The Assessment of Psychosocial Distress in Hospitalized Cancer Patients During Radio‐Oncological Treatment: A Monocentric Experience Study.” Supportive Care in Cancer 32, no. 12: 785. 10.1007/s00520-024-08977-3. 39535622

[brb370282-bib-0063] Marino, K. , M. Goodberlet , and P. Cyrus . 2021. “Review of Pharmacologic Sleep Agents for Critically Ill Patients.” Critical Care Nursing Clinics of North America 33, no. 2: 145–153. 10.1016/j.cnc.2021.01.006. 34023082

[brb370282-bib-0064] McNicholas, W. T. 1993. “Sleep and Medical Disorders.” Irish Medical Journal 86, no. 6: 183–184. 8106222

[brb370282-bib-0065] Medic, G. , M. Wille , and M. E. H. Hemels . 2017. “Short‐ and Long‐Term Health Consequences of Sleep Disruption.” Nature and Science of Sleep 9: 151–161. 10.2147/NSS.S134864 PMC5449130 28579842

[brb370282-bib-0066] Meyer, T. J. , S. E. Eveloff , M. S. Bauer , W. A. Schwartz , N. S. Hill , and R. P. Millman . 1994. “Adverse Environmental Conditions in the Respiratory and Medical ICU Settings.” Chest 105, no. 4: 1211–1216. 10.1378/chest.105.4.1211. 8162751

[brb370282-bib-0067] Miettinen, T. , J. Sverloff , O. P. Lappalainen , S. J. Linton , K. Sipilä , and E. Kalso . 2022. “Sleep Problems in Pain Patients Entering Tertiary Pain Care: The Role of Pain‐Related Anxiety, Medication Use, Self‐Reported Diseases, and Sleep Disorders.” Pain 163, no. 7: E812–E820. 10.1097/j.pain.0000000000002497. 34561395 PMC9199106

[brb370282-bib-0068] Miller, M. A. , B. N. Renn , F. Chu , and N. Torrence . 2019. “Sleepless in the Hospital: A Systematic Review of Non‐Pharmacological Sleep Interventions.” General Hospital Psychiatry 59: 58–66. 10.1016/j.genhosppsych.2019.05.006. 31170567 PMC6620136

[brb370282-bib-0069] Nilius, G. , M. Richter , and M. Schroeder . 2021. “Updated Perspectives on the Management of Sleep Disorders in the Intensive Care Unit.” Nature and Science of Sleep 13: 751–762. 10.2147/NSS.S284846. PMC8200142 34135650

[brb370282-bib-0070] Oksenberg, A. , and D. S. Silverberg . 2009. “Avoiding the Supine Posture During Sleep for Patients With Mild Obstructive Sleep Apnea.” American Journal of Respiratory and Critical Care Medicine 180, no. 1: 101. 10.1164/ajrccm.180.1.101. 19535668

[brb370282-bib-0071] Oseni, T. I. A. , N. E. Udonwa , A. O. Oku , M. T. Makinde , and F. Archibong . 2024. “Association Between Sleep Quality and Blood Pressure Control Among Hypertensive Patients at a Rural Tertiary Hospital in Southern Nigeria: A Cross‐Sectional Study.” BMJ Open 14, no. 3: e079774. 10.1136/bmjopen-2023-079774. PMC10928733 38458777

[brb370282-bib-0072] Ouellet, M. C. , and S. M. C. Beaulieu‐Bonneau . 2019. “Traumatic Brain injury.” In Handbook of Sleep Disorders in Medical Conditions, 221–252. Academic Press, Cambridge, MA.

[brb370282-bib-0073] Pamuk, K. , and N. Turan . 2022. “The Effect of Light on Sleep Quality and Physiological Parameters in Patients in the Intensive Care Unit.” Applied Nursing Research 66: 151607. 10.1016/j.apnr.2022.151607. 35840273

[brb370282-bib-0074] Parthasarathy, S. , and M. J. Tobin . 2002. “Effect of Ventilator Mode on Sleep Quality in Critically III Patients.” American Journal of Respiratory and Critical Care Medicine 166, no. 11: 1423–1429. 10.1164/rccm.200209-999OC. 12406837

[brb370282-bib-0075] Patel, A. K. , V. Reddy , and J. F. Araujo . 2020. “Physiology, Sleep Stages.” StatPearls 3: 1–4. 30252388

[brb370282-bib-0076] Pek, E. A. , A. Remfry , C. Pendrith , C. Fan‐Lun , R. S. Bhatia , and C. Soong . 2017. “High Prevalence of Inappropriate Benzodiazepine and Sedative Hypnotic Prescriptions Among Hospitalized Older Adults.” Journal of Hospital Medicine 12, no. 5: 310–316. 28459898 10.12788/jhm.2739

[brb370282-bib-0077] Rampes, S. , K. Ma , Y. A. Divecha , A. Alam , and D. Ma . 2020. “Postoperative Sleep Disorders and Their Potential Impacts on Surgical Outcomes.” Journal of Biomedical Research 34, no. 4: 271–280. 10.7555/JBR.33.20190054. PMC7386412 32519977

[brb370282-bib-0078] Roehrs, T. R. T. 2023. The Effects of Medications on Sleep Quality and Sleep Architecture. In Uptodate. Wolters Kluwer, Netherlands.

[brb370282-bib-0079] Ruettgers, N. , A. C. Naef , M. Rossier , et al. 2022. “Perceived Sounds and Their Reported Level of Disturbance in Intensive Care Units: A Multinational Survey Among Healthcare Professionals.” PLoS One 17, no. 12: e0279603. 10.1371/journal.pone.0279603. 36584079 PMC9803129

[brb370282-bib-0080] Salahub, C. , P. E. Wu , L. D. Burry , et al. 2023. “Melatonin for Insomnia in Medical Inpatients: A Narrative Review.” Journal of Clinical Medicine 12, no. 1: 256. 10.3390/jcm12010256. PMC9821578 36615056

[brb370282-bib-0081] Shelgikar, A. V. , and R. Chervin . 2013. “Approach to and Evaluation of Sleep Disorders.” Continuum: Lifelong Learning in Neurology 19, no. 1: 32–49. 10.1212/01.CON.0000427214.00092.0f. 23385693

[brb370282-bib-0082] Showler, L. , Y. Ali Abdelhamid , J. Goldin , and A. M. Deane . 2023. “Sleep During and Following Critical Illness: A Narrative Review.” World Journal of Critical Care Medicine 12, no. 3: 92–115. 10.5492/wjccm.v12.i3.92. 37397589 PMC10308338

[brb370282-bib-0083] Smith, M. G. , M. Cordoza , and M. Basner . 2022. “Environmental Noise and Effects on Sleep: An Update to the WHO Systematic Review and Meta‐Analysis.” Environmental Health Perspectives 130, no. 7: 76001. 10.1289/EHP10197. 35857401 PMC9272916

[brb370282-bib-0084] Stewart, N. H. , and V. M. Arora . 2022. “Sleep in Hospitalized Older Adults.” Sleep Medicine Clinics 17, no. 2: 223–232. 10.1016/j.jsmc.2022.02.002. 35659075

[brb370282-bib-0085] Stewart, N. H. , and V. M. Arora . 2022. “Sleep in Hospitalized Patients.” Clocks Sleep 130: 151–165. 10.1007/978-3-030-93739-3_20. PMC7509688 33089161

[brb370282-bib-0086] Tan, X. , L. van Egmond , M. Partinen , T. Lange , and C. Benedict . 2019. “A Narrative Review of Interventions for Improving Sleep and Reducing Circadian Disruption in Medical Inpatients.” Sleep Medicine 59: 42–50. 10.1016/j.sleep.2018.08.007. 30415906

[brb370282-bib-0087] Toublanc, B. , D. Rose , J. C. Glérant , et al. 2007. “Assist‐Control Ventilation vs. Low Levels of Pressure Support Ventilation on Sleep Quality in Intubated ICU Patients.” Intensive Care Medicine 33, no. 7: 1148–1154. 17492431 10.1007/s00134-007-0659-2

[brb370282-bib-0088] Vaishnav, P. P. , A. Suresh , S. Kooragayalu , and S. Kooragayalu . 2023. “Sleep Disturbances in Hospitalized and Intensive Care Unit Patients.” In Sleep Apnea Frontiers. Progress in Sleep Research, edited by A. S. BaHammam and M. Hunasikatti , pp. 231–253. Springer, Singapore. 10.1007/978-981-99-7901-1_15.

[brb370282-bib-0089] van den Ende, E. S. , K. D. I. van Veldhuizen , B. Toussaint , et al. 2021. “Hospitalized COVID‐19 Patients Were Five Times More Likely to Suffer from Total Sleep Deprivation Compared to Non‐COVID‐19 Patients; an Observational Comparative Study.” Frontiers in Neuroscience 15, no. 1: 680932. 10.3389/fnins.2021.680932. 34675762 PMC8525610

[brb370282-bib-0090] Waheed, Z. , and U. Kalsoom . 2024. “The Prevalence of Anxiety, Depression, and Sleep Disturbances among the Rheumatoid Arthritis Patients.” Journal of Postgraduate Medical Institute 38, no. 1: 54–58. 10.54079/jpmi.38.1.3282.

[brb370282-bib-0091] Wang, J. , N. Wu , and L. Zhang . 2022. “The Causal Relationship Between Sleep and Obesity: Novel Insights and Therapeutic Target.” Journal of Clinical Endocrinology and Metabolism 107, no. 10: E4265–E4266. 10.1210/clinem/dgac372. 35715884 PMC9516120

[brb370282-bib-0092] Wesselius, H. M. , E. S. Van Den Ende , J. Alsma , et al. 2018. “Quality and Quantity of Sleep and Factors Associated With Sleep Disturbance in Hospitalized Patients.” JAMA Internal Medicine 178, no. 9: 1165–1171. 10.1001/jamainternmed.2018.2669. 30014139 PMC6142965

[brb370282-bib-0093] White, B. , H. S. Snyder , and M. V. B. Patel . 2023. “Evaluation of Medications Used for Hospitalized Patients with Sleep Disturbances: A Frequency Analysis and Literature Review.” Journal of Pharmacy Practice 36, no. 1: 126–138. 10.1177/08971900211017857. 34096384

[brb370282-bib-0094] Yetton, B. D. , E. A. McDevitt , N. Cellini , C. Shelton , and S. C. Mednick . 2018. “Quantifying Sleep Architecture Dynamics and Individual Differences Using Big Data and Bayesian Networks.” PLoS One 13, no. 4: e0194604. 10.1371/journal.pone.0194604. 29641599 PMC5894981

[brb370282-bib-0095] Young, J. S. , J. A. Bourgeois , D. M. Hilty , and K. A. Hardin . 2009. “Sleep in Hospitalized Medical Patients, Part 2: Behavioral and Pharmacological Management of Sleep Disturbances.” Journal of Hospital Medicine 4, no. 1: 50–59. 19140196 10.1002/jhm.397

[brb370282-bib-0096] Yun, J. Y. , C. Y. Lee , and H. Kim . 2024. “Effects of Sleep Hygiene on Sleep Disturbance in Patients With Parkinsonism.” Parkinsonism & Related Disorders 122: 106621. 10.1016/j.parkreldis.2024.106621.

[brb370282-bib-0097] Zhang, Y. , Zhao J hao , and Huang D ya , et al. 2020. “Multiple Comorbid Sleep Disorders Adversely Affect Quality of Life in Parkinson's Disease Patients.” Npj Parkinson's Disease 6: 106621. 10.1038/s41531-020-00126-x. PMC7492275 33015354

