# Correction to “Optimizing Sleep Disorder Management in Hospitalized Patients: Practical Approach for Healthcare Providers”

**DOI:** 10.1002/brb3.70678

**Published:** 2025-07-27

Roostaei, G., N. Khoshnam Rad, B. Rahimi, et al. 2025, Optimizing Sleep Disorder Management in Hospitalized Patients: Practical Approach for Healthcare Providers. Brain and *Behavior*, 15: e70282. https://doi.org/10.1002/brb3.70282

In the originally published version, the heading levels were misnumbered throughout. The online version has been updated accordingly.

We apologize for this error.

